# Intravitreal S100B Injection Leads to Progressive Glaucoma Like Damage in Retina and Optic Nerve

**DOI:** 10.3389/fncel.2018.00312

**Published:** 2018-09-26

**Authors:** Sandra Kuehn, Wilhelm Meißner, Pia Grotegut, Carsten Theiss, H. Burkhard Dick, Stephanie C. Joachim

**Affiliations:** ^1^Experimental Eye Research Institute, University Eye Hospital, Ruhr-University Bochum, Bochum, Germany; ^2^Department of Cytology, Institute of Anatomy, Ruhr-University Bochum, Bochum, Germany

**Keywords:** glaucoma, animal model, S100B protein, retinal ganglion cell damage, optic nerve degeneration, electroretinogram

## Abstract

The glial protein S100B, which belongs to a calcium binding protein family, is up-regulated in neurological diseases, like multiple sclerosis or glaucoma. In previous studies, S100B immunization led to retinal ganglion cell (RGC) loss in an experimental autoimmune glaucoma (EAG) model. Now, the direct degenerative impact of S100B on the retina and optic nerve was evaluated. Therefore, 2 μl of S100B was intravitreally injected in two concentrations (0.2 and 0.5 μg/μl). At day 3, 14 and 21, retinal neurons, such as RGCs, amacrine and bipolar cells, as well as apoptotic mechanisms were analyzed. Furthermore, neurofilaments, myelin fibers and axons of optic nerves were evaluated. In addition, retinal function and immunoglobulin G (IgG) level in the serum were measured. At day 3, RGCs were unaffected in the S100B groups, when compared to the PBS group. Later, at days 14 and 21, the RGC number as well as the β-III tubulin protein level was reduced in the S100B groups. Only at day 14, active apoptotic mechanisms were noted. The number of amacrine cells was first affected at day 21, while the bipolar cell amount remained comparable to the PBS group. Also, the optic nerve neurofilament structure was damaged from day 3 on. At day 14, numerous swollen axons were observed. The intraocular injection of S100B is a new model for a glaucoma like degeneration. Although the application site was the eye, the optic nerve degenerated first, already at day 3. From day 14 on, retinal damage and loss of function was noted. The RGCs in the middle part of the retina were first affected. At day 21, the damage expanded and RGCs had degenerated in all areas of the retina as well as amacrine cells. Furthermore, elevated IgG levels in the serum were measured at day 21, which could be a sign of a late and S100B independet immune response. In summary, S100B had a direct destroying impact on the axons of the optic nerve. The damage of the retinal cell bodies seems to be a consequence of this axon loss.

## Introduction

The causes of the most retina degenerations are unidentified, this includes glaucoma. Glaucoma is defined by a progressive loss of retinal ganglion cells (RGCs) and degeneration of the optic nerve, which leads to slow continuous loss of the visual field (EGS, 2017). The pathomechanisms of this disease are still unknown. Oxidative stress (Tezel et al., 2010), excitotoxicity (Dreyer et al., 1996) and immunological processes (Grus et al., 2004) might play a role. An approach of the last years was to study of the association of the immune system and autoimmune processes with glaucoma. It could be demonstrated that glaucoma patients have altered autoantibody titers, both up- and down-regulated, against different types of structural molecules and proteins, such as heat shock proteins (Tezel et al., 1998; Joachim et al., 2007), phosphatidylserine (Kremmer et al., 2001), γ-enolase (Maruyama et al., 2000), neuron specific enolase (Ikeda et al., 2002) and glutathione-S-transferase (Yang et al., 2001). Altered antibody pattern were detected not only in serum, but also in aqueous humor or tear fluid of glaucoma patients. An increase of autoreactive antibodies against the S100B protein was detectable in the tears of glaucoma patients (Grus et al., 2010).

S100B is a small calcium binding protein, which is expressed in glia cells (Huttunen et al., 2000). It belongs to the S100 protein family, which currently consists of 25 members (Marenholz et al., 2006; Santamaria-Kisiel et al., 2006), and represents a potential effector of inflammatory reactions and oxidative stress in neurons (Huttunen et al., 2000; Rothermundt et al., 2003). Furthermore, it is also known that increased concentrations of S100B occur in neurodegenerative diseases, like Alzheimer’s disease (Goncalves et al., 2000), epilepsy (Rothermundt et al., 2001) and schizophrenia (Griffin et al., 1989). The relevance of the protein S100B in pathophysiology of glaucoma was also investigated in an animal model. In this case, the S100B was applied systemically in rats and induced an immune response against S100B. Due to this damage mechanism, this model is called experimental autoimmune glaucoma model (EAG model). The optic nerve filament degenerated at first (Noristani et al., 2016), while the neurons of the inner retinal layers were affected later in the EAG model (Reinehr et al., 2018). S100B directly influences pathophysiological mechanisms inducing glaucomatous degenerations in retina and optic nerve.

In order to examine the local effects of the S100B protein on retina and optic nerve and to exclude potentially systemically immunological effects, we applied S100B intravitreally in rat eyes. The goal of this study is to investigate, if it is possible to ascertain a RGCs deprivation and optic nerve degeneration after intravitreal application of S100B.

## Materials and Methods

### Animals

Eight week old male Wistar rats from Charles River (176–200 g; Sulzfeld, Germany) were included in this study. All experiments that involved animals were performed in compliance with the ARVO Statement for the Use of Animals in Ophthalmic and Vision Research. Furthermore, the study was approved by the animal care committee of North Rhine-Westphalia in Germany (84-02.04.2013.A442). During the whole experiment, the rats were kept in a room with constant temperature and a 12:12 h dark-light lightening cycle. They were housed under environmentally controlled conditions with free access to food and water *ad libitum* and in the absence of pathogens.

### Intraocular S100B Injection

Rats were anesthetized with a mixture of ketamine (50 mg/ml, Ratiopharm) and xylazine (2%, Bayer Health Care). After the application of a topical anesthetic (Conjuncain, 4 mg/ml, Bausch&Lomb), the pupil was dilated with a mydriaticum (Tropicamide, 5 mg/ml, Stulln). The S100B protein was used in two different concentrations. One group was treated with 2 μl of a 0.2 μg/μl S100B solution (S100B I group, Sigma-Aldrich) and the other group was treated with 2 μl of a 0.5 μg/μl S100B solution (S100B II group, Sigma-Aldrich). S100B was injected in the vitreous of one eye with a 32-gauge needle (Hamilton) under a stereomicroscope (Zeiss). The control group received 2 μl PBS (Biochrome), since this was used as a solvent for S100B. The corresponding eyes remained untreated. Therefore, four groups were compared in this study: native, PBS, S100B I and S100B II. After the injection, the eyes were treated with Floxal, an antibiotic ointment (Bausch&Lomb), and examined after 2 h and on the next day. Animals with eye bleeding or cataracts were excluded. In addition, the general behavior and look of the animals was evaluated once a week.

### Intraocular Pressure Measurements

The intraocular pressure (IOP) of all groups (*n* = 6 eyes/group) was measured using a rebound tonometer (TonoLab; Icare, Oy, Finland) up to day 21, once a week (Biermann et al., 2012). Means were calculated from 10 single measurements per eye and point in time.

### Electroretinogram Analysis

The rats were dark adapted for the electroretinogram (ERG) recording overnight. The measurements were done under dark conditions. A headlamp with red light was needed for the orientation of the investigator. A full-field flash electroretinography (HMsERG system; OcuScience LLC, Rolla, MO, USA) was used to analyze the function of the retina at day 14 (Schmid et al., 2014). Therefore, rats were anesthetized with a ketamine/xylazine mixture (100/4 mg/kg). Eyes were dilated with a mydriaticum and locally anesthetized with conjuncain. During the measurement the body temperature was maintained at 37°C with a temperature controller (TC-1000, CWE Inc., Ardmore, PA, USA). A ground electrode was placed subcutaneously in the back over the tail and reference electrodes were located under the both ears. One drop of methocel (Omni Vision, Puchheim, Germany) was directly applicated on the cornea. Recording electrodes combined with a contact lens were placed in the center of both eyes. The function of the electrodes was tested before the faraday cage was closed over the whole equipment (OcuScience, LLC). Scotopic flash series were recorded with light flash intensity at 0.1, 0.3, 1, 3, 10 and 25 cd/m^2^. The ERGView 4.380R software (OcuScience, LLC) was used for the data evaluation. Therefore, a low pass filter (150 Hz) was necessary. All single waves were controlled. The amplitude of the a- and b-wave was exported to excel (Microsoft Corp., Redmond, WA, USA) for further statistical analysis.

### IgG ELISA Measurement

The immunoglobulin G (IgG) level was measured in the sera of PBS and S100B I animals at 14 and 21 days. Doublets of undiluted serum sample were analyzed using an IgG ELISA kit (Cloud-Clone Corp.) according to the manufacture’s protocol. All measurements were performed on a microplate reader (AESKU Reader with Gen5 ELISA Software, AESKU. DIAGNOSTICS). The IgG level of the S100B I was compared to the level of the PBS group at day 14 and 21 (*n* = 7/group and point in time).

### Immunohistochemistry of Retinal Flatmounts After 3 and 14 Days

Three and 14 days after the intraocular injection of PBS or S100B, eyes were prepared for flatmounts as previously described (Casola et al., 2015; 3 days: *n* = 3–4/group; 14 days: *n* = 6–7/group). Briefly, after fixation in 4% paraformaldehyde (PFA; Merck) for 2 h, the eye was opened and the retina was gently dissected. The retina was divided into four sections and then mounted on a black gridded nitrocellulose membrane (Millipore) with the vitreous facing up. For investigation of the RGCs, the retinas were stained with an antibody against Brn-3a (Table 1) in a 12-well plate on a shaker. With an Apotom.2 microscope (Zeiss) four images per flatmount arm (one peripheral, two middle and one central photo) were taken. The Brn-3a^+^ cells were counted in a masked fashion and the percentage of Brn-3a^+^ cells was calculated by setting the mean count of the native group to 100%.

**Table 1:** Primary and secondary antibodies used for immune histological and Western blot stainings.

Primary antibodies			Secondary antibodies		
Name	Company	Dilution	Name	Company	Dilution
**Retinal flatmounts**					
Brn-3a	Santa Cruz	1:100	Donkey anti-goat AlexaFluor 488	Abcam	1:400
**Retinal cross-sections**					
Brn-3a	Santa Cruz	1:100	Donkey anti-goat AlexaFluor 488	Abcam	1:400
Calretinin	Millipore	1:2,000	Donkey anti-goat AlexaFluor 488	Abcam	1:500
PKCα	Santa Cruz	1:300	Donkey anti-mouse A488	Invitrogen	1:500
**Longitudinal optic nerve sections**					
SMI-32	Biolegend	1:2,000	Goat anti-mouse A488	Invitrogen	1:400
**Western blot**					
β-actin	Cell Signaling	1:1,000	Donkey anti-rabbit DyeLight800	Thermo Fisher	1:20,000
β-actin	Sigma-Aldrich	1:6,000	IRDye donkey anti-mouse DL800	LICOR	1:20,000
β-III-tubulin	R&D System	1:15,000	Donkey anti-mouse AlexaFluor 680	Invitrogen	1:5,000
Calretinin	Millipore	1:30,000	Rabbit anti-goat AlexaFluor 680	Invitrogen	1:5,000
Cleaved caspase 3	Sigma-Aldrich	1:300	Donkey anti-rabbit AlexaFluor 680	Invitrogen	1:5,000
PARP	Abcam	1:400	Donkey anti-rabbit AlexaFluor 680	Invitrogen	1:5,000

### Preparation of Retina Cross-Section and Optic Nerve Longitudinal Sections

Eyes and optic nerves were examined 3, 14 and 21 days after intraocular injection of PBS and S100B, like previously described (Noristani et al., 2016). Subsequently, eyes (14 and 21 days: 6–7 retinas/group) were fixed with 4% PFA for 1 h and finally treated with 30% sucrose. Thereafter, retinal cross-sections (10 μm) were cut with a microtome (Thermo Scientific, Waltham, MA, USA). Optic nerves were fixed for 2 h in 4% PFA followed by 30% sucrose and cut in 4 μm thick longitudinal sections (3 days: 3–4 optic nerves/group; 14 and 21 days: 6–7 optic nerves/group). The retinal cross-sections as well as optic nerve longitudinal sections were mounted on slices (Histobond). Finally, the tissues on the slices were fixed in ice-cold acetone for 10 min.

### Histological Stainings and Analysis

Hematoxylin and eosin (HE, both Merck) staining was used for retinal cross-sections (Horstmann et al., 2013). We microscopically examined (Axio Imager.M1, Zeiss) the HE stained retinal cross-sections and recorded four images per retina (two peripheral and two central).

Luxol fast blue (LFB, RAL Diagnostic) staining of optic nerve sections was performed (Noristani et al., 2016). Microscopy and image acquisition of the LFB stained sections was carried out by recording three pictures per nerve (proximal, middle and distal). The pictures were scored from 0 = intact up to 2 = destroyed, in regard to integrity of the nerve fibers. The LFB scoring focuses on the shorting of myelin fibers and fissure development in the “combed” structure (Horstmann et al., 2013). The statistical evaluation of the LFB score was performed by a comparative analysis of the S100B groups with the native as well as with the PBS treated group.

### Immunofluorescence Staining of Specific Cell Types in Retina and Optic Nerve

The staining protocol was performed as previously described (Noristani et al., 2016). Briefly, retinal cross-sections and optic nerve longitudinal sections were blocked with a mixture of a 10%–20% serum, 0.1%–0.2% TritonX-100 (Sigma-Aldrich) and PBS (Santa Cruz). The primary antibodies (Table 1) were diluted in the same mixture and incubated at room temperature overnight. Then, sections were incubated with AlexaFluor 555 or AlexaFluor 488 labeled secondary antibodies in the same mixture (Table 1). The nuclei were stained with DAPI (0.01 μg, Sera) for 5 min. Finally, sections were covered with Shandon-mount (Thermo Scientific, Waltham, MA, USA). Negative controls of all stainings were performed using secondary antibodies only.

For further evaluations, four images per retina (two peripheral and two central) and three per optic nerve (proximal, middle and distal) were taken with an Axio Imager.M1 microscope (Zeiss) in 400× magnification. The cell quantification of retinas was carried out by counting the positive signals (Brn-3a, calretinin, protein kinase Cα (PKCα)). We used an already established scoring system for the analysis of SMI-32 labeled neurofilaments in optic nerves (Noristani et al., 2016). The SMI-32 scoring respects the shortening of the neurofilaments and the development of retraction bulbs (0 = intact, 2 = destroyed).

### Preparation of Optic Nerve Cross-Sections

At day 14, animals (*n* = 6–7/group) were perfused with 4% PFA solution for 20 min. Thereafter, the optic nerves were fixed in 2.5% glutaraldehyde in 1% phosphate buffer (PB). A detailed description of the embedding procedure is given in Krause et al. (2016). Briefly, after rinsing in PB the optic nerves were placed in Dalton solution (5% potassium dichromate, 3.4% sodium chloride, distilled water and osmium tetra-oxide) and then dehydrated in an ethanol series. The tissue was carefully transferred into epoxy resin and followed by an EPON mixture using a slightly modified protocol first described by Luft (1961). 0.5 μm thick semi-thin slices were cut with a Leica EM TRIM (Leica Microsystems GmbH, Wetzlar, Germany).

Methylene blue stained cross-sections (*n* = 6/optic nerve) were scored as described by Pang and Clark (2007), which reach from 1 (intact) up to 5 (damage with strong gliosis and axon swelling up to 95% of optic nerve).

### Western Blot Analysis

For Western blot analysis we prepared retinas as previously described (Noristani et al., 2016). Initially, proteins had to be isolated from the tissue. Therefore, the retinas were homogenized with mechanical (metal homogenizer, Neolab) and chemical methods (150 μl of the commercial lysis buffer RIPA, Cell Signaling Technology). After the samples were incubated on ice for 50 min, then centrifugation took place and the supernatants were pipetted off. We determined the protein concentration in the samples with a light absorption photometry. For the gel electrophoresis we used 12% Bis-Tris or 4%–12% Bis-Tris gels (NuPAGE, Invitrogen) and loaded 10 μg sample per lane. After performing the blotting with NuPAGE transfer buffer (Invitrogen), the nitrocellulose membranes were blocked with a mixture of 5% milk powder (Applichem) in a PBS/0.05% Tween-20 solution (Santa Cruz, Sigma-Aldrich). For the detection of the different proteins we used specific primary antibodies (Table 1). For the detection of the bound primary antibodies fluorochrome labeled compatible secondary antibodies were used (Table 1). The visualization and analysis of the protein bands was done by means of the Odyssey infrared imager system 2.1 (LI-COR Biosciences). For the statistical evaluation, the band intensities of the target protein were always normalized with β-actin and then the normalized intensities of the S100B group were evaluated against both normalized controls.

### Statistics

The statistical analysis was performed using Statistica software (version 13, Dell). Data are presented as mean ± standard error (SEM). In the course of the statistical analysis, the S100B groups were compared with the respective PBS (*) and native group (^#^) using one-way ANOVA with *post hoc* Tukey test. Student’s *t-test* was used to compare both groups of the ELISA analysis. A *p* < 0.05 was considered statistically significant.

## Results

### Unchanged Intraocular Pressure With First Signs of Disrupted Signal Transfer

**Figure 1:**
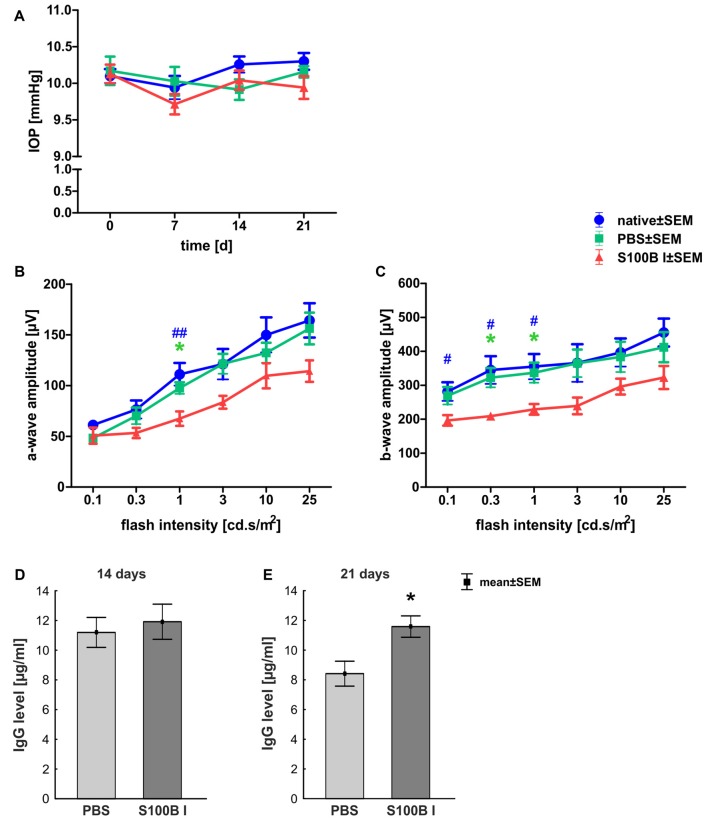
Stable intraocular pressure (IOP) and reduced electroretinogram (ERG) amplitudes. **(A)** The IOP was measured weekly for 21 days. No differences between the groups was noted at all points in time. **(B)** The a-wave amplitude of the ERG measurements was only affected at 1.0 cd.s/m^2^ in the S100B I group (PBS: *p* = 0.049, native: *p* = 0.004). **(C)** However, the b-wave amplitude of the S100B I group was reduced compared to the PBS group at 0.3 (*p* = 0.035) and 1.0 cd.s/m^2^ (*p* = 0.04) and in comparison to the native group at 0.1 cd (*p* = 0.048), 0.3 cd (*p* = 0.01) and 1.0 cd (*p* = 0.02). **(D)** The immunoglobulin G (IgG) level in the sera of PBS and S100B I group was analyzed via IgG ELISA. No differences between both groups were measured at day 14. **(E)** Later, at day 21, the IgG level in the serum of the S100B I group was higher than that of the PBS group (*p* = 0.014). *p < 0.05 (compared to PBS group); ^#^*p* < 0.05, ^##^*p* < 0.01 (compared to native group).

IOP was measured 1 day before S100B was intravitreally injected and 7, 14 and 21 days after the injection. At all points in time, the IOP of the S100B I group remained comparable to the PBS (*p* > 0.05) and native group (*p* > 0.05, Figure 1A).

The a- and b-wave amplitudes of the retina were investigated via ERG. The a-wave amplitude of the S100B I group was slightly reduced at a flash intensity of 1 cd.s/m^2^ (67.5 ± 7.1 μV) compared to the PBS (97.5 ± 5.7 μV, *p* = 0.049) and to the native group (111.1 ± 11.1 μV, *p* = 0.004; Figure 1B). No differences were noted between all groups at all other flash intensities. The b-wave amplitude of the S100B I group was decreased at 0.1 cd (196.5 ± 15.4 μV), 0.3 cd (209.2 ± 11.0 μV) and 1 cd (41.7 ± 15.8 μV) compared to the native group (0.1 cd: 281.5 ± 27.2 μV, *p* = 0.048; 0.3 cd: 345.1 ± 40.7 μV, *p* = 0.01; 1 cd: 355.1 ± 37.2 μV, *p* = 0.02; Figure 1C). In comparison to the PBS group, only the flash intensities at 0.3 (322.5 ± 28.3 μV, *p* = 0.035) and 1 cd (337.0 ± 29.5 μV, *p* = 0.04) were altered.

The IgG level was analyzed in the sera of PBS and S100B I group. At day 14, the PBS group had a mean IgG level of 11.2 ± 1.0 μg/ml. A similar IgG level was noted in S100B I serum samples (11.9 ± 1.2 μg/ml, *p* = 0.65, Figure 1D). However, higher IgG level were measured in the S100B I group (11.6 ± 0.7 μg/ml) in comparison to the PBS group (8.4 ± 0.8 μg/ml, *p* = 0.014, Figure 1E) at day 21.

### Analysis of Retina

#### Intact Retina Structure Despite Time-Dependent Apoptotic Mechanisms

**Figure 2:**
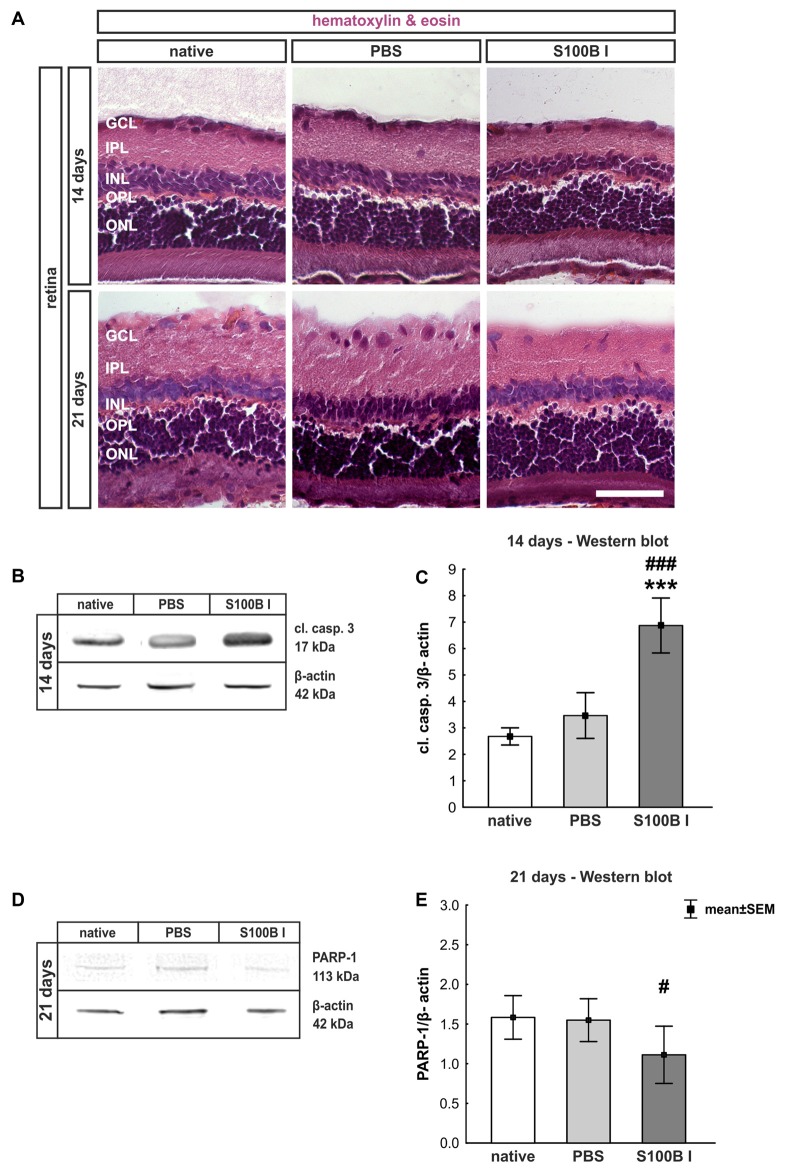
Intact retinal morphology, but peak of apoptosis at day 14. **(A)** Via hematoxylin and eosin staining, we could show that retinas still had an intact structure at 14 and 21 days in all groups. S100B I did not induce cellular infiltrations or damage to retinal layers. **(B)** Western blot analysis of cleaved caspase 3 (17 kDa) was normalized with β-actin (42 kDa) at 14 days. **(C)** At day 14, the protein level of cleaved caspase 3 was increased in the S100B I group compared to PBS and native group (both: *p* < 0.001). **(D)** At day 21, poly (ADP-ribose) polymerase 1 (PARP-1; 113 kDa) was analyzed via Western blot. **(E)** Here, differences between the S100B I and native group were observed (*p* = 0.027) and a decreasing trend in comparison to the PBS group (*p* = 0.06). GCL, ganglion cell layer; IPL, inner plexiform layer; INL, inner nuclear layer; OPL, outer plexiform layer; ONL, outer nuclear layer; scale bar = 50 μm; ***p < 0.001 (compared to PBS group); ^#^*p* < 0.05, ^###^*p* < 0.001 (compared to native group).

In order to gain a first impression, we explored the effects of S100B on retinal cross-sections and evaluated the HE stained sections according to morphological aspects at day 14 and 21. The retina layers and structure of all groups remained intact during the course of the study (Figure 2A). No signs of retinal inflammation were noted. However, apoptotic mechanisms occurred in the S100B I group. Western blot analysis of cleaved caspase 3 (Figure 2B) showed an increased signal intensity in the S100B I group (7.6 ± 0.9 units) compared to the PBS (2.8 ± 0.7 units, *p* < 0.001) and native group (2.4 ± 0.1 units, *p* < 0.001, Figure 2C) at day 14. At day 21, Western blot stainings of poly (ADP-ribose) polymerase 1 (PARP-1) were performed (Figure 2D). A reduction of PARP-1 at 113 kDa is a sign for apoptosis (Yu et al., 2006). A decreasing trend of PARP-1 level was observed in the S100B I group (0.8 ± 0.3 units) compared to the PBS group (1.8 ± 0.2 units, *p* = 0.06; Figure 2E). In comparison to the native group (1.9 ± 0.3 units), a lower protein level was noted in S100B retinas (*p* = 0.027, Figure 2E).

#### Time-Dependent but Concentration Independent Loss of Retinal Ganglion Cells

**Figure 3:**
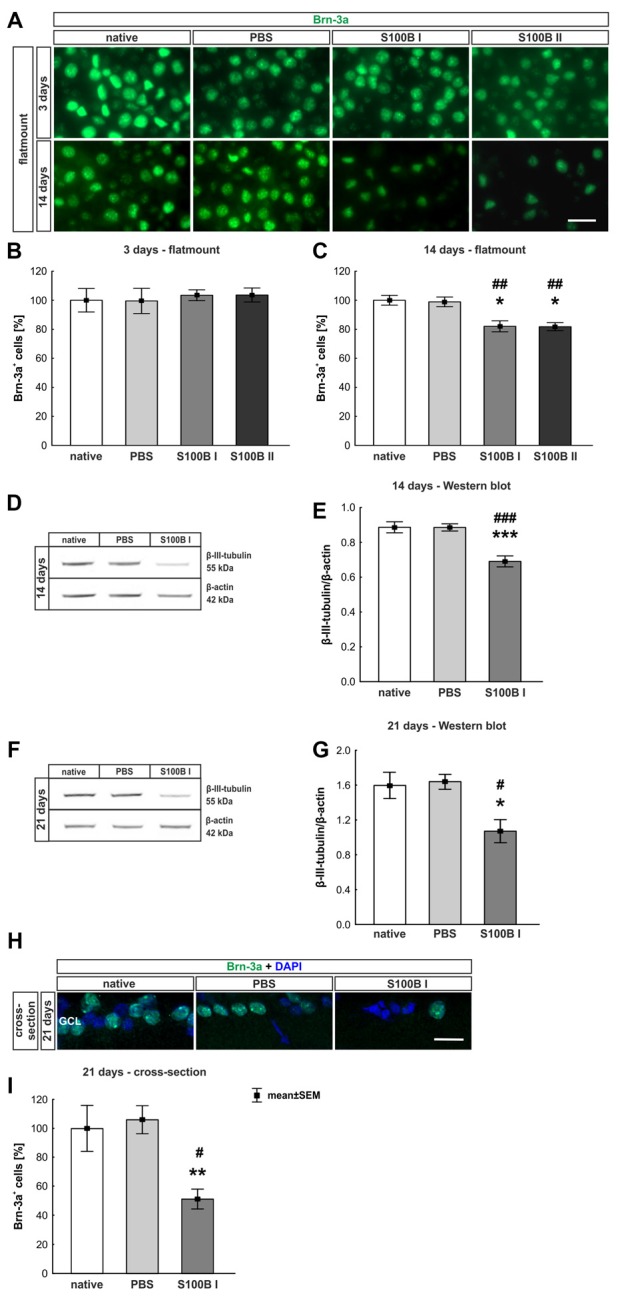
Significant retinal ganglion cell (RGC) loss from day 14 on. **(A)** RGCs were stained on retinal flatmounts with anti-Brn-3a antibody at 3 and 14 days. **(B)** After 3 days, RGC density in the S100B I and II group was comparable to the PBS and native group. **(C)** However, after 14 days, a significant reduction of RGC density was noticed in both S100B groups compared to the PBS (both: *p* = 0.01) and native group (S100B I: *p* = 0.008, S100B II: *p* = 0.003). **(D)** Western blots of β-III-tubulin (55 kDa) were analyzed using β-actin (42 kDa) for normalization at day 14. **(E)** A significant reduction of β-III-tubulin signal intensity was noted in the S100B I group compared to both controls (both: *p* < 0.001). **(F)** Also, at day 21, the β-III-tubulin protein level was analyzed with Western blot. **(G)** Here, a significant reduction of the signal intensity in the S100B I group could be seen in comparison to PBS and native group (both: *p* = 0.02). **(H)** RGCs were stained with Brn-3a on retinal cross-section at day 21. **(I)** A degeneration of RGCs was seen in the S100B I group compared to the PBS (*p* = 0.006) and native group (*p* = 0.02). GCL, ganglion cell layer; scale bar = 20 μm, *p < 0.05, **p < 0.01, ***p < 0.001 (compared to PBS group); ^#^*p* < 0.05, ^##^*p* < 0.01, ^###^*p* < 0.001 (compared to native group).

Three and 14 days after the intraocular application of S100B in two different concentrations retinal flatmounts were stained with anti-Brn-3a (Figure 3A). At day 3, the Brn-3a^+^ cell counts of the native (100.0 ± 8.1% Brn-3a^+^ cells, *p* > 0.9) and PBS group (99.5 ± 8.7% Brn-3a^+^ cells) were similar to counts of the S100B I (103.5 ± 3.7%, *p* > 0.9) and S100B II group (103.6 ± 4.8%, *p* > 0.9, Figure 3B) in all evaluated areas (Supplementary Figure S1A). However, a reduced Brn-3a^+^ cell number was noted in both S100B groups (S100B I: 82.1 ± 3.8%, S100B II: 81.8 ± 2.8%) compared to the PBS (98.9 ± 3.3%, both *p* = 0.01) and native group (100.0 ± 3.4%, S100B I: *p* = 0.008, S100B II: *p* = 0.003, Figure 3C) at day 14. In relation to the PBS group (101.3 ± 3.6%) the degeneration started in the middle part of the retina in both S100B concentrations (S100B I: 83.6 ± 3.5%, *p* = 0.009; S100B II: 80.6 ± 2.6%, *p* = 0.002, Supplementary Figure S1B). Similar findings were observed in comparison to the native group (100.0 ± 3.5%; S100B I: *p* = 0.02; S100B II: *p* = 0.002). The loss of RGC was independent of the applied S100B concentration, so subsequent evaluations were done with the lower S100B concentration.

In addition to immunohistochemistry, we investigated the influence of S100B on the neurons via Western blot analysis and detected β-III-tubulin, a specific marker for neurons (Figures 3D,F). The evaluation underlined the immune histological results, since the β-III-tubulin intensity was significantly lower in the S100B I group (0.69 ± 0.03 units) in comparison to the PBS (0.89 ± 0.02 units; *p* < 0.001) and native group (0.89 ± 0.03 units; *p* < 0.001, Figure 3E) at 14 days. We evaluated the effects of the intraocular application of S100B I at 21 days. Here, a lower β-III-tubulin signal intensity was also measured in the S100B I group (1.08 ± 0.35 units) when compared to the PBS (1.65 ± 0.08 units, *p* = 0.02) or native group (1.61 ± 0.15 units, *p* = 0.02, Figure 3G).

To verify the Western blot results at day 21, retinal cross-sections were stained with an anti-Brn-3a antibody (Figure 3H). Fewer Brn-3a^+^ cells were noted in the S100B I (16.53 ± 12.61 cells/mm) than in the PBS (34.29 ± 3.21 cells/mm, *p* = 0.006) and native group (32.36 ± 5.15 cells/mm, *p* = 0.02, Figure 3I). We could prove the first degenerations signs of the RGCs at day 14. This was independently of the S100B concentration. The damage aggravated at day 21.

#### Significant Degeneration of Amacrine Cells After S100B Injection

**Figure 4:**
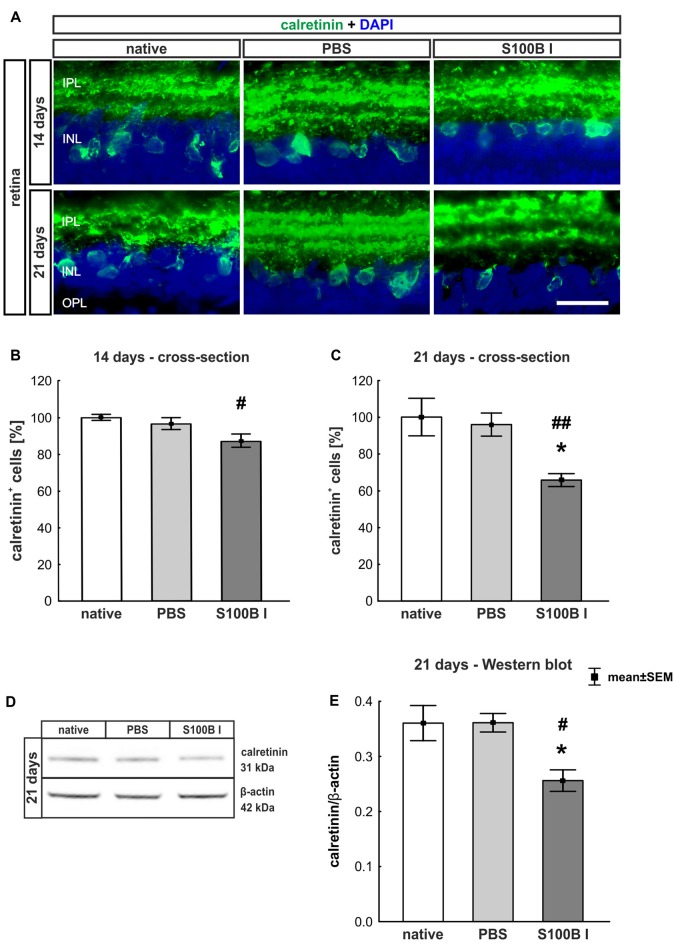
Progressive loss of amacrine cells after intraocular S100B injection. **(A)** We performed immunohistochemical evaluations of amacrine cells (calretinin, green) 14 and 21 days after S100B injection. Cell nuclei were stained with DAPI (blue). **(B)** The examination of calretinin^+^ cells at 14 days revealed a trend to a loss of cell bodies compared to the PBS (*p* = 0.084) and a lower cell number compared to the native group (*p* = 0.015). **(C)** Whereas, the evaluation after 21 days already showed significantly fewer amacrine cells in the S100B I group compared to the both controls (PBS: *p* = 0.014, native: *p* = 0.0098). **(D)** The protein level of calretinin (31 kDa) was measured with Western blot at day 21 and normalized with β-actin (42 kDa). **(E)** The calretinin protein level decreased at day 21 in comparison to both controls (both: *p* = 0.025). IPL, inner plexiform layer; INL, inner nuclear layer; scale bar = 20 μm, *p < 0.05 (compared to PBS group); ^#^*p* < 0.05, ^##^*p* < 0.01 (compared to native group).

In order to evaluate the extent of retinal damage after the intraocular application of S100B, the cells of the inner nuclear layer (INL) were analyzed. For this purpose, we performed immune histology using an anti-calretinin antibody, a specific marker for amacrine cells (Figure 4A). The count of calretinin^+^ cells showed a tendency of reduced cell numbers in the S100B I group (87.07 ± 9.58%) compared to the PBS group (96.48 ± 8.58%, *p* = 0.084) and a lower cell count compared to the native group after 14 days (100.0 ± 1.64%, *p* = 0.015, Figure 4B). This damage extended later on, since we could observe a significant loss of amacrine cells in the S100B I group (65.71 ± 9.26%), compared to both control groups, PBS (95.91 ± 16.64%, *p* = 0.014) and native retinas (100.0 ± 10.2%, *p* = 0.0098, Figure 4C), after 21 days. This result was confirmed by Western blot analysis (Figure 4D). Calretinin signal was significantly reduced in the S100B I group (0.26 ± 0.06 units) compared to the PBS group (0.35 ± 0.043 units, *p* = 0.025) and to the native group (0.35 ± 0.03 units, *p* = 0.025; Figure 4E) at day 21.

#### Preserved Integrity of Bipolar Cells

**Figure 5:**
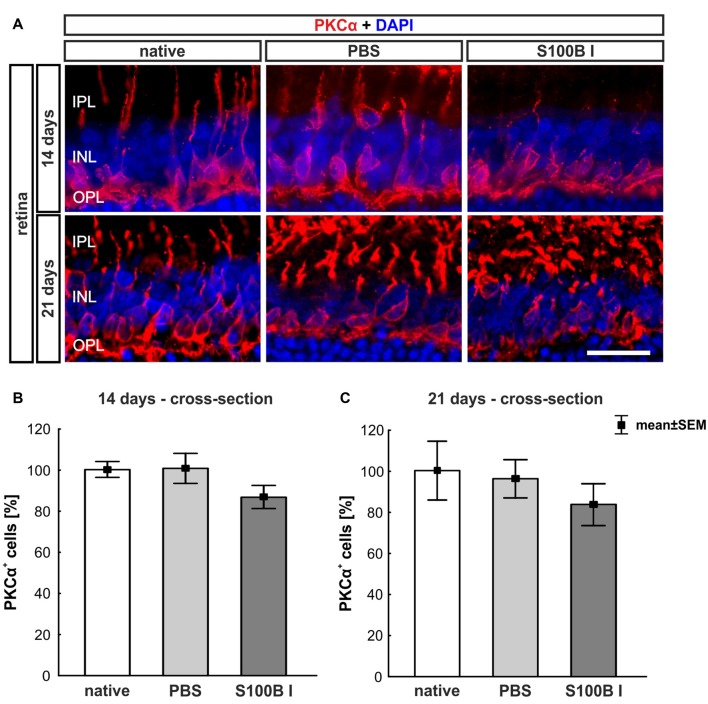
No effect on bipolar cells. **(A)** Exemplary images of retinal cross-sections with bipolar cell (protein kinase Cα, PKCα, red) and cell nuclei (DAPI, blue) staining were shown. **(B)** The PKCα^+^ cell count was comparable in all groups at day 14. **(C)** A similar result was seen at day 21. IPL, inner plexiform layer; INL, inner nuclear layer; OPL, outer plexiform layer; scale bar = 20 μm.

To analyze a possible degeneration of other neurons of the INL, bipolar cells were examined on retina section with an antibody against PKCα, a specific marker for bipolar cells (Figure 5A). PKCα^+^ cell counts did not show any alterations in the S100B I group at 14 (PBS: 100.47 ± 7.2%, S100B I: 86.47 ± 5.54%; *p* = 0.17, Figure 5B) and 21 days (PBS: 95.96 ± 9.32%, S100B I: 83.46 ± 10.21% *p* = 0.63, Figure 5C).

### Analysis of Optic Nerve

#### Tendency of Slow Progressive Degeneration of Myelin Sheaths

**Figure 6:**
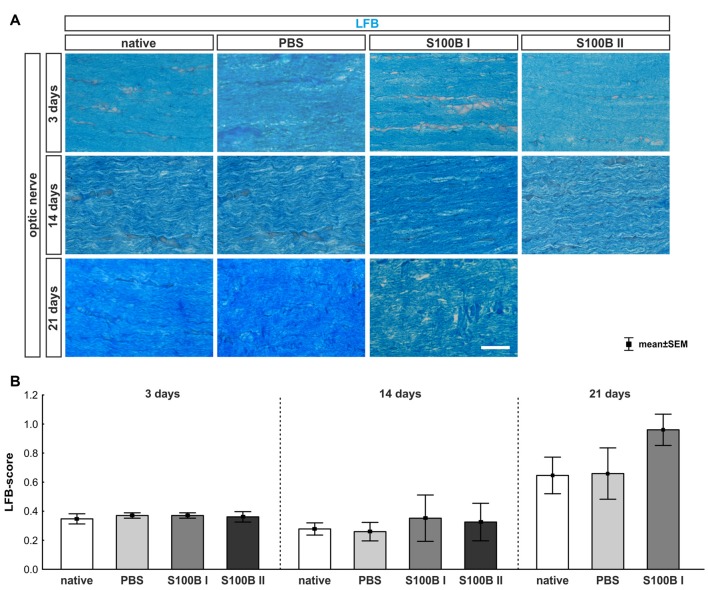
Intact optic nerve myelin sheaths in all groups. **(A)** Luxol fast blue (LFB) was used to stain the optic nerve myelin sheath. **(B)** At day 3, the optic nerves did not show any degeneration. This was also observed at day 14. However, after 21 days, a trend towards an increased LFB score was recorded in the S100B I group when compared to the native group (*p* = 0.22), but not when compared to the PBS group. Scale bar = 20 μm.

We examined longitudinal optic nerve sections 3, 14 and 21 days after the intravitreal application of S100B. We labeled myelin sheaths with LFB (Figure 6A) to score the grade of demyelination. Intact myelin sheaths were noted at day 3 in the S100B I (0.37 ± 0.02, *p* > 0.9) and S100B II group (0.36 ± 0.04, *p* > 0.9) compared to the PBS (0.37 ± 0.02) and to the native group (0.34 ± 0.03, Figure 6B). Similar results could be seen at day 14. The myelin sheaths of the optic nerves showed no affect for both applied S100B concentrations (S100B I: 0.35 ± 0.16, *p* > 0.8, S100B II: 0.32 ± 0.13, *p* > 0.9, Figure 6B) when compared to the PBS group (0.27 ± 0.06) and to the native group (0.27 ± 0.04). After 21 days, a tendency to alteration of the optic nerve sheaths was noted in the S100B I group (0.96 ± 0.11) compared to the PBS group (0.66 ± 0.17, *p* = 0.22, Figure 6B). No differences to the native group were observed (0.64 ± 0.13, *p* = 0.38).

#### Progressive Destruction of Optic Nerve Neurofilaments

**Figure 7:**
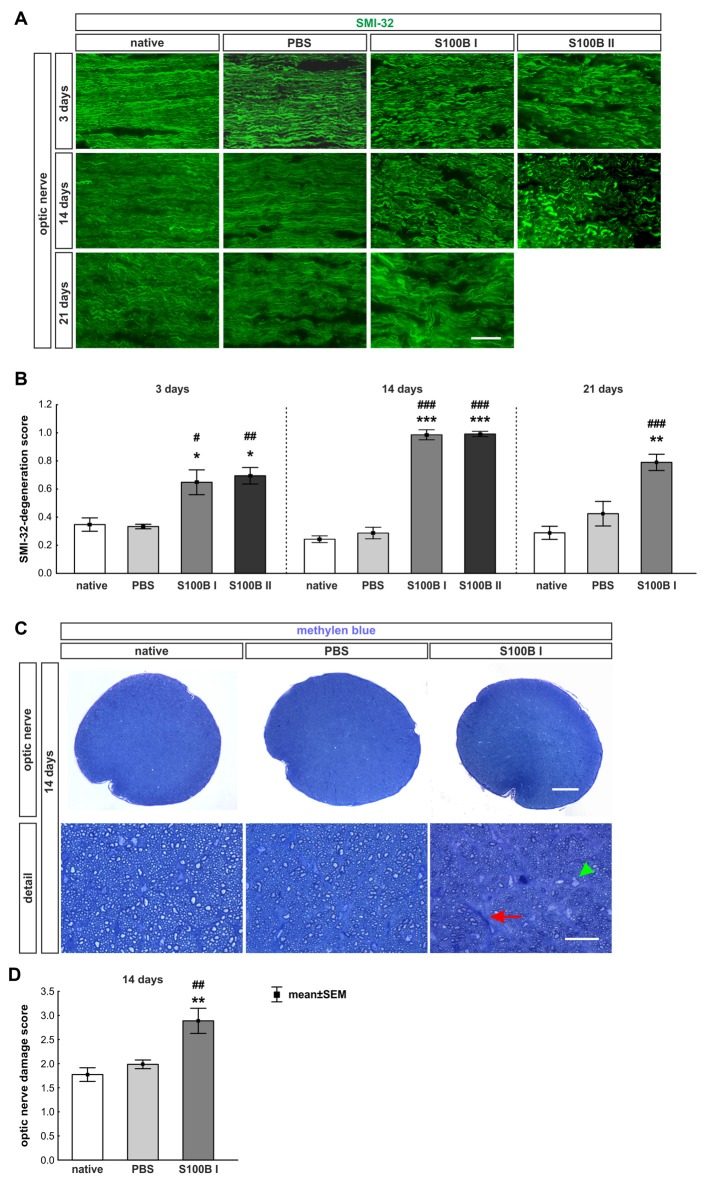
S100B induced progressive degeneration of optic nerve neurofilaments. **(A)** We analyzed neurofilaments of longitudinal optic nerve sections at 3, 14 and 21 days with anti-SMI-32-antibody (green). **(B)** At day 3, the neurofilament showed a low grade of degeneration in the S100B I (PBS: *p* = 0.02, native: *p* = 0.03) and S100B II group (PBS: *p* = 0.01, native: *p* = 0.005). These degeneration signs aggravated after 14 days compared to PBS and native group (both controls: S100B I: *p* < 0.001, S100B II: *p* < 0.001). After 21 days, destruction of neurofilaments was stable in S100B I group in comparison to PBS (*p* = 0.003) and native group (*p* < 0.001). **(C)** In addition, methylene blue stained cross-sections were scored at day 14. **(D)** The S100B I optic nerves showed more gliosis and an increased number of swollen axons compared to the PBS (*p* = 0.007) and native group (*p* = 0.001). Scale bar: **(A)** = 20 μm, **(C)** = 100 μm, **(C)** detail = 20 μm, red arrow: gliosis, green arrow head: swollen axon; *p < 0.05; **p < 0.01; ***p < 0.001 (compared to PBS group); ^#^*p* < 0.05; ^##^*p* < 0.01; ^###^*p* < 0.001 (compared to native group).

The labeling of neurofilaments with an antibody to SMI-32 was performed for a more specific evaluation of the optic nerve structure (Figure 7A). S100B induced damage to the neurofilaments from day 3 on (S100B I: 0.65 ± 0.09, *p* = 0.02, S100B II: 0.69 ± 0.06, *p* = 0.01) compared to the PBS group (0.34 ± 0.02). In comparison to the native group (0.35 ± 0.05) similar results were noticed in the S100B I (*p* = 0.03) and the S100B II group (*p* = 0.005, Figure 7B). These alterations of the SMI-32 stained structure were progressive. At 14 days, the integrity and structure of the neurofilaments in both S100B groups (S100B I: 0.99 ± 0.04, *p* < 0.001, S100B II: 0.99 ± 0.02, *p* < 0.001) was strongly degenerated in comparison to the PBS group (0.29 ± 0.04, Figure 7B). In comparison to the native group (0.24 ± 0.02), the neurofilament damage in both S100B groups was again very prominent (both: *p* < 0.001). We also examined the neurofilaments after 21 days, where the level of destruction remained stable (PBS: 0.42 ± 0.09, S100B I: 0.79 ± 0.06, *p* = 0.003). Comparison of the S100B I and native group (0.29 ± 0.05) showed a similar result in regard to the level of degeneration (*p* < 0.001, Figure 7B).

Furthermore, methylene blue stained optic nerve cross-sections were scored (Figure 7C). The structures of the native (1.77 ± 0.14) and the PBS group (1.99 ± 0.09, *p* = 0.7) were comparable. However, the S100B I injection (2.89 ± 0.26) increased the gliosis signs and the number of swollen axons compared to the PBS (*p* = 0.007) and native group (*p* = 0.001, Figure 7D). The density of intact axons decreased, especially in the middle part of the S100B I optic nerves.

## Discussion

The intravitreal injection of S100B led to an dose independent, but time-dependent degeneration of retina and optic nerve. At day 3, a slight damage of the optic nerve was noted, but the retina was not yet affected. Therefore, we assume the degeneration processes started in the optic nerve. Later, at day 14, RGCs and their axons were damaged via apoptotic mechanisms. However, the myelin fibers remained intact. At the later point in time, further progress of damage was observed. Amacrine cells were affected in the retina from day 21 on and also the optic nerve myeline sheath showed first signs of dissolution. In addition, increased IgG levels in the serum were noted later on.

### Impact of S100B on the Retina

It is known that S100B affects neurons in a dose-dependent manner. In a cortical neuronal cell culture or in *in vitro* models, nanomolare doses of S100B protected neurons (Villarreal et al., 2011), while micromolare levels had deleterious effects (Donato, 2001). In our study, S100B was applied in two different doses, 400 nmol and 1 μmol. Both doses showed comparable destroying effects on RGCs in this rodent model. Hence, we did not observe any An dose-dependent effect and only used the lower S100B dose for further investigations.

### Time-Dependent Damage of Retinal Ganglion Cells and Optic Nerve

In previous studies, a systemic immunization with S100B first induced a wallerian-like degeneration of the optic nerve (Noristani et al., 2016). Meaning, the axons are initially damaged, while the myelin structure remains intact (Saggu et al., 2010). In this EAG model, the decline of RGC numbers followed later on (Casola et al., 2015; Noristani et al., 2016). A similar degeneration progression was also observed in an ocular hypertension model (Son et al., 2010).

The initial site of damage in this new model is of great interest, since S100B was applied locally to the eye. With our novel approach, we could also observe a wallerian-like degeneration of the optic nerve, which occurred earlier than the RGC loss. Also, other intravitreally applicated substances, like kainic acid, induce a wallerian-like degeneration of the optic nerve (Massoll et al., 2013). The intravitreal injection of TNF-α also led at first to an axon loss with a subsequent retrograde retinal cell body damage at later damage stages (Kitaoka et al., 2006). However, the intravitreal injection of other substances, such as N-methyl-D-aspartate (NMDA; Kuehn et al., 2017) first affected the structures of the retina, while the damage of the optic nerve was a consequence of the cell body loss in the retina.

One explanation for the different site of action for S100B could be that it is mainly expressed by astrocytes (Petrova et al., 2000; Ponath et al., 2007; Brozzi et al., 2009), which have their origin in the optic nerve (Holländer et al., 1991). The receptors or further binding structures for the other intravitreal injected substances are directly situated in the retina. However, both, RGCs and their axons, degenerated earlier in our study than in the EAG model. The intravitreal S100B injection acted directly and did not need to activate an immune response first, which takes more time. The late findings of elevated IgG levels in the serum undermine the conclusion that the intravitreal S100B injection did not trigger a systemic immune response by themself. A possible cause for the late systemic immune response in this new model could be the enhanced damage in retina and optic nerve, which then damaged the blood-retina barrier. A brake-down of this barrier was for example described for a ocular hypertension animal model (Trost et al., 2015). The blood-retina barrier usually controls the immigration of immune cells in the retina (Reichenbach and Bringmann, 2013). One of these cells are B-cells. To produce IgGs B-cells need a priming contact with the antigen and an antigen presenting cell (Janeway, 2002). A porous blood-retina barrier could led to this contact. Previous studys, using the EAG model, could detected alterations of the systemic auto-antibodies patterns (Joachim et al., 2009) and retinal IgG deposits (Joachim et al., 2012). It is postulated that the blood-retina barrier in patients is damaged during glaucoma and therefore auto-antibody pattern alterations were also be detected systemically (Joachim et al., 2005). Furthermore, IgG autoantibody accumulations and B-cells were observed in human retinas (Gramlich et al., 2013). Meaning, a strong retinal damage could trigger an immune response which strengthen the degeneration.

S100B is a protein which regulates the intracellular calcium level (Donaldson et al., 1995; Donato, 2001). Another intravitreally used molecule, which led to an influx of calcium ions in cells, is NMDA. It binds directly to the NMDA receptor, which opens the calcium channels in uncontrolled manner (Urushitani et al., 2001). S100B might bind to the receptor for advanced glycation endproducts (RAGE; Villarreal et al., 2011). In comparison, the degeneration progression through NMDA is faster than through S100B. One day after NMDA injection, RGCs began to degenerate (Manabe and Lipton, 2003) and after 3 days RGCs in the middle and peripheral part of the retina were lost (Kuehn et al., 2017). S100B needs more time for a similar damage pattern. We did not note degeneration signs at 3 days. At day 14, only the RGCs in the middle part of the retina were affected and then at day 21 all regions of the retina showed cell loss.

### Only Inner Retinal Layers Were Affected

S100B is a protein, which is more associated with astrocytes than with Müller glia in the retina (Petrova et al., 2000; Brozzi et al., 2009). This could also be confirmed in our study. The astrocytes in this model were mostly located from the nerve fiber layer (NFL) to the INL. These layers are the only layers, which were affected in the current study. Not only the RGC but also the amacrine cell number declined, while rod bipolar cell numbers remained stable. Possibly, the astrocytes were triggered in a destroying manner through the S100B application (Villarreal et al., 2014). Then, reactive astrocytes have a extensive molecular repertoire which were involved in all cellular aspects (Sofroniew, 2009). The amacrine cell number decline could also have another cause. Amacrine cells and RGCs are connected via gap junctions and they could transmit the cell death signal (Akopian et al., 2016). The time-cause of the cell loss in this model seems to confirm this assumption, since RGCs were affected from day 14 on and the amacrine cells later, at day 21. However, the functionality of the cells in the INL was alreadey negatively affected at day 14, before cell numbers decreased.

### Time-Dependent Apoptotic Mechanisms

Apoptosis is an early hallmark of degeneration in retina damage models (Wang et al., 2005; Noristani et al., 2016). It is known that a high level of S100B can activate the caspase cascade (Donato, 2001). In this study, we noted an increase of cleaved caspase 3, a prominent apoptotic protein, at day 14, which can be induced via intrinsic and extrinsic apoptotic pathways (Nair et al., 2014). At day 21, we detected PARP-1, since this protein will be cleaved by the caspase 3 (D’Amours et al., 2001), which is a late apoptotic mechansism. We could measure a trend for a decline of PARP-1. These results are hints for terminated apoptotic processes at day 21.

## Conclusion

We could demonstrate, for the first time, that a local, intravitreal injection of S100B leads to glaucoma like damage. Although the application site was the eye, the optic nerve degenerated first, already at day 3. From day 14 on, retinal damage was noted. The RGCs in the middle part of the retina were first affected. At day 21, the damage expanded. RGCs and amacrine cells degenerated in all parts of the retina. Thence, S100B had a direct destroying impact on the axons of the optic nerve, which then led to the damage of the retinal cell bodies.

## Author Contributions

SK designed the study, carried out experiments, performed statistical analyses, drafted the manuscript and generated graphics. WM carried out experiments, performed statistical analyses and revised the manuscript. PG carried out experiments and generated graphics. CT carried out experiments and revised the manuscript. HD revised the manuscript. SJ designed the study and drafted the manuscript. All authors read and approved the final manuscript.

## Conflict of Interest Statement

The authors declare that the research was conducted in the absence of any commercial or financial relationships that could be construed as a potential conflict of interest. The reviewer TA and the handling Editor declared their shared affiliation, at the time of the review.

## Supplementary Material

FIGURE S1 RGC density in different flatmount regions. **(A)** At day 3, the RGC numbers in all three regions, peripheral, middle and central of S100B flatmounts were comparable to the PBS and native group. **(B)** At day 14, RGC loss was noted in the middle part of the retina compared to the PBS (S100B I: *p* = 0.009, S100B II: *p* = 0.002) and native group (S100B I: *p* = 0.02, S100B II: *p* = 0.002). ***p* < 0.01 (compared to PBS group), ^#^*p* < 0.05, ^##^*p* < 0.01 (compared to native group).

## Abbreviations

EAG model: experimental autoimmune glaucoma model
ERG: electroretinogram
GCL: ganglion cell layer
HE: hematoxylin and eosin
IgG: immunoglobulin G
INL: inner nuclear layer
IOP: intraocular pressure
IPL: inner plexiform layer
LFB: luxol fast blue
NFL: nerve fiber layer
NMDA: N-methyl-D-aspartate
ONL: outer nuclear layer
OPL: outer plexiform layer
PARP-1: poly (ADP-ribose) polymerase 1
PB: phosphate buffer
PFA: paraformaldehyde
PKCα: protein kinase Cα
RGC: retinal ganglion cell
SEM: standard error of mean.

## References

[B1] Akopian A. Kumar S. Ramakrishnan H. Viswanathan S. Bloomfield S. A. (2016). Amacrine cells coupled to ganglion cells via gap junctions are highly vulnerable in glaucomatous mouse retinas. J. Comp. Neurol. [Epub ahead of print]. 10.1002/cne.24074 27411041 PMC7047713

[B2] Biermann J. van Oterendorp C. Stoykow C. Volz C. Jehle T. Boehringer D.. (2012). Evaluation of intraocular pressure elevation in a modified laser-induced glaucoma rat model. Exp. Eye Res. 104, 7–14. 10.1016/j.exer.2012.08.011 22981807

[B3] Brozzi F. Arcuri C. Giambanco I. Donato R. (2009). S100B protein regulates astrocyte shape and migration via interaction with Src kinase: implications for astrocyte development, activation, and tumor growth. J. Biol. Chem. 284, 8797–8811. 10.1074/jbc.M805897200 19147496 PMC2659238

[B4] Casola C. Schiwek J. E. Reinehr S. Kuehn S. Grus F. H. Kramer M.. (2015). S100 alone has the same destructive effect on retinal ganglion cells as in combination with HSP 27 in an autoimmune glaucoma model. J. Mol. Neurosci. 56, 228–236. 10.1007/s12031-014-0485-2 25577368

[B5] D’Amours D. Sallmann F. R. Dixit V. M. Poirier G. G. (2001). Gain-of-function of poly(ADP-ribose) polymerase-1 upon cleavage by apoptotic proteases: implications for apoptosis. J. Cell Sci. 114, 3771–3778. 11707529 10.1242/jcs.114.20.3771

[B6] Donaldson C. Barber K. R. Kay C. M. Shaw G. S. (1995). Human S100b protein: formation of a tetramer from synthetic calcium-binding site peptides. Protein Sci. 4, 765–772. 10.1002/pro.5560040416 7613474 PMC2143092

[B7] Donato R. (2001). S100: a multigenic family of calcium-modulated proteins of the EF-hand type with intracellular and extracellular functional roles. Int. J. Biochem. Cell Biol. 33, 637–668. 10.1016/s1357-2725(01)00046-2 11390274

[B8] Dreyer E. B. Zurakowski D. Schumer R. A. Podos S. M. Lipton S. A. (1996). Elevated glutamate levels in the vitreous body of humans and monkeys with glaucoma. Arch. Ophthalmol. 114, 299–305. 10.1001/archopht.1996.01100130295012 8600890

[B9] EGS. (2017). European glaucoma society terminology and guidelines for glaucoma, 4th edition—chapter 2: classification and terminology supported by the EGS foundation part 1: foreword; introduction; Glossary; Chapter 2 classification and terminology. Br. J. Ophthalmol. 101, 73–127. 10.1136/bjophthalmol-2016-egsguideline.002 28424171 PMC5583685

[B10] Goncalves D. S. Lenz G. Karl J. Goncalves C. A. Rodnight R. (2000). Extracellular S100B protein modulates ERK in astrocyte cultures. Neuroreport 11, 807–809. 10.1097/00001756-200003200-00030 10757524

[B11] Gramlich O. W. Beck S. von Thun Und Hohenstein-Blaul N. Boehm N. Ziegler A. Vetter J. M.. (2013). Enhanced insight into the autoimmune component of glaucoma: IgG autoantibody accumulation and pro-inflammatory conditions in human glaucomatous retina. PLoS One 8:e57557. 10.1371/journal.pone.0057557 23451242 PMC3581473

[B12] Griffin W. S. Stanley L. C. Ling C. White L. MacLeod V. Perrot L. J.. (1989). Brain interleukin 1 and S-100 immunoreactivity are elevated in Down syndrome and Alzheimer disease. Proc. Natl. Acad. Sci. U S A 86, 7611–7615. 10.1073/pnas.86.19.7611 2529544 PMC298116

[B13] Grus F. H. Boehm N. Beck S. Schlich M. Lossbrandt U. Pfeiffer N. (2010). Autoantibody profiles in tear fluid as a diagnostic tool in glaucoma. Invest. Ophthalmol. Vis. Sci. 51:6110.

[B14] Grus F. H. Joachim S. C. Hoffmann E. M. Pfeiffer N. (2004). Complex autoantibody repertoires in patients with glaucoma. Mol. Vis. 10, 132–137. 14990890

[B15] Holländer H. Makarov F. Dreher Z. van Driel D. Chan-Ling T. L. Stone J. (1991). Structure of the macroglia of the retina: sharing and division of labour between astrocytes and Müller cells. J. Comp. Neurol. 313, 587–603. 10.1002/cne.903130405 1783683

[B16] Horstmann L. Schmid H. Heinen A. P. Kurschus F. C. Dick H. B. Joachim S. C. (2013). Inflammatory demyelination induces glia alterations and ganglion cell loss in the retina of an experimental autoimmune encephalomyelitis model. J. Neuroinflammation 10:120. 10.1186/1742-2094-10-120 24090415 PMC3851328

[B17] Huttunen H. J. Kuja-Panula J. Sorci G. Agneletti A. L. Donato R. Rauvala H. (2000). Coregulation of neurite outgrowth and cell survival by amphoterin and S100 proteins through receptor for advanced glycation end products (RAGE) activation. J. Biol. Chem. 275, 40096–40105. 10.1074/jbc.M006993200 11007787

[B18] Ikeda Y. Maruyama I. Nakazawa M. Ohguro H. (2002). Clinical significance of serum antibody against neuron-specific enolase in glaucoma patients. Jpn. J. Ophthalmol. 46, 13–17. 10.1016/s0021-5155(01)00455-5 11853708

[B19] Janeway C. A. (Ed.). (2002). Immunologie. Heidelberg, Berling: Spektrum Verlag.

[B20] Joachim S. C. Bruns K. Lackner K. J. Pfeiffer N. Grus F. H. (2007). Antibodies to α B-crystallin, vimentin and heat shock protein 70 in aqueous humor of patients with normal tension glaucoma and IgG antibody patterns against retinal antigen in aqueous humor. Curr. Eye Res. 32, 501–509. 10.1080/02713680701375183 17612966

[B21] Joachim S. C. Gramlich O. W. Laspas P. Schmid H. Beck S. von Pein H. D.. (2012). Retinal ganglion cell loss is accompanied by antibody depositions and increased levels of microglia after immunization with retinal antigens. PLoS One 7:e40616. 10.1371/journal.pone.0040616 22848388 PMC3406064

[B22] Joachim S. C. Grus F. H. Kraft D. White-Farrar K. Barnes G. Barbeck M.. (2009). Complex antibody profile changes in an experimental autoimmune glaucoma animal model. Invest. Ophthalmol. Vis. Sci. 50, 4734–4742. 10.1167/iovs.08-3144 19458332

[B23] Joachim S. C. Pfeiffer N. Grus F. H. (2005). Autoantibodies in patients with glaucoma: a comparison of IgG serum antibodies against retinal, optic nerve, and optic nerve head antigens. Graefes Arch. Clin. Exp. Ophthalmol. 243, 817–823. 10.1007/s00417-004-1094-5 15834611

[B24] Kitaoka Y. Kitaoka Y. Kwong J. M. Ross-Cisneros F. N. Wang J. Tsai R. K.. (2006). TNF-α-induced optic nerve degeneration and nuclear factor-kappaB p65. Invest. Ophthalmol. Vis. Sci. 47, 1448–1457. 10.1167/iovs.05-0299 16565378

[B25] Krause M. Brune M. Theiss C. (2016). Preparation of human formalin-fixed brain slices for electron microscopic investigations. Ann. Anat. 206, 27–33. 10.1016/j.aanat.2016.04.030 27136748

[B26] Kremmer S. Kreuzfelder E. Klein R. Bontke N. Henneberg-Quester K. B. Steuhl K. P.. (2001). Antiphosphatidylserine antibodies are elevated in normal tension glaucoma. Clin. Exp. Immunol. 125, 211–215. 10.1046/j.1365-2249.2001.01578.x 11529911 PMC1906120

[B27] Kuehn S. Rodust C. Stute G. Grotegut P. Meißner W. Reinehr S.. (2017). Concentration-dependent inner retina layer damage and optic nerve degeneration in a NMDA model. J. Mol. Neurosci. 63, 283–299. 10.1007/s12031-017-0978-x 28963708

[B28] Luft J. H. (1961). Improvements in epoxy resin embedding methods. J. Biophys. Biochem. Cytol. 9, 409–414. 10.1083/jcb.9.2.409 13764136 PMC2224998

[B29] Manabe S. Lipton S. A. (2003). Divergent NMDA signals leading to proapoptotic and antiapoptotic pathways in the rat retina. Invest. Ophthalmol. Vis. Sci. 44, 385–392. 10.1167/iovs.02-0187 12506100

[B30] Marenholz I. Lovering R. C. Heizmann C. W. (2006). An update of the S100 nomenclature. Biochim. Biophys. Acta 1763, 1282–1283. 10.1016/j.bbamcr.2006.07.013 16938360

[B31] Maruyama I. Ohguro H. Ikeda Y. (2000). Retinal ganglion cells recognized by serum autoantibody against γ-enolase found in glaucoma patients. Invest. Ophthalmol. Vis. Sci. 41, 1657–1665. 10845582

[B32] Massoll C. Mando W. Chintala S. K. (2013). Excitotoxicity upregulates SARM1 protein expression and promotes Wallerian-like degeneration of retinal ganglion cells and their axons. Invest. Ophthalmol. Vis. Sci. 54, 2771–2780. 10.1167/iovs.12-10973 23518770 PMC3632266

[B33] Nair P. Lu M. Petersen S. Ashkenazi A. (2014). Apoptosis initiation through the cell-extrinsic pathway. Methods Enzymol. 544, 99–128. 10.1016/b978-0-12-417158-9.00005-4 24974288

[B34] Noristani R. Kuehn S. Stute G. Reinehr S. Stellbogen M. Dick H. B.. (2016). Retinal and optic nerve damage is associated with early glial responses in an experimental autoimmune glaucoma model. J. Mol. Neurosci. 58, 470–482. 10.1007/s12031-015-0707-2 26746422

[B35] Pang I. H. Clark A. F. (2007). Rodent models for glaucoma retinopathy and optic neuropathy. J. Glaucoma 16, 483–505. 10.1097/ijg.0b013e3181405d4f 17700292

[B36] Petrova T. V. Hu J. Van Eldik L. J. (2000). Modulation of glial activation by astrocyte-derived protein S100B: differential responses of astrocyte and microglial cultures. Brain Res. 853, 74–80. 10.1016/s0006-8993(99)02251-9 10627310

[B37] Ponath G. Schettler C. Kaestner F. Voigt B. Wentker D. Arolt V.. (2007). Autocrine S100B effects on astrocytes are mediated via RAGE. J. Neuroimmunol. 184, 214–222. 10.1016/j.jneuroim.2006.12.011 17254641

[B38] Reichenbach A. Bringmann A. (2013). New functions of Muller cells. Glia 61, 651–678. 10.1002/glia.22477 23440929

[B39] Reinehr S. Kuehn S. Casola C. Koch D. Stute G. Grotegut P.. (2018). HSP27 immunization reinforces AII amacrine cell and synapse damage induced by S100 in an autoimmune glaucoma model. Cell Tissue Res. 371, 237–249. 10.1007/s00441-017-2710-0 29064077

[B40] Rothermundt M. Missler U. Arolt V. Peters M. Leadbeater J. Wiesmann M.. (2001). Increased S100B blood levels in unmedicated and treated schizophrenic patients are correlated with negative symptomatology. Mol. Psychiatry 6, 445–449. 10.1038/sj.mp.4000889 11443531

[B41] Rothermundt M. Peters M. Prehn J. H. Arolt V. (2003). S100B in brain damage and neurodegeneration. Microsc. Res. Tech. 60, 614–632. 10.1002/jemt.10303 12645009

[B42] Saggu S. K. Chotaliya H. P. Blumbergs P. C. Casson R. J. (2010). Wallerian-like axonal degeneration in the optic nerve after excitotoxic retinal insult: an ultrastructural study. BMC Neurosci. 11:97. 10.1186/1471-2202-11-97 20707883 PMC2930628

[B43] Santamaria-Kisiel L. Rintala-Dempsey A. C. Shaw G. S. (2006). Calcium-dependent and -independent interactions of the S100 protein family. Biochem. J. 396, 201–214. 10.1042/bj20060195 16683912 PMC1462724

[B44] Schmid H. Renner M. Dick H. B. Joachim S. C. (2014). Loss of inner retinal neurons after retinal ischemia in rats. Invest. Ophthalmol. Vis. Sci. 55, 2777–2787. 10.1167/iovs.13-13372 24699380

[B45] Sofroniew M. V. (2009). Molecular dissection of reactive astrogliosis and glial scar formation. Trends Neurosci. 32, 638–647. 10.1016/j.tins.2009.08.002 19782411 PMC2787735

[B46] Son J. L. Soto I. Oglesby E. Lopez-Roca T. Pease M. E. Quigley H. A.. (2010). Glaucomatous optic nerve injury involves early astrocyte reactivity and late oligodendrocyte loss. Glia 58, 780–789. 10.1002/glia.20962 20091782

[B47] Tezel G. Seigel G. M. Wax M. B. (1998). Autoantibodies to small heat shock proteins in glaucoma. Invest. Ophthalmol. Vis. Sci. 39, 2277–2287. 9804136

[B48] Tezel G. Yang X. Luo C. Kain A. D. Powell D. W. Kuehn M. H.. (2010). Oxidative stress and the regulation of complement activation in human glaucoma. Invest. Ophthalmol. Vis. Sci. 51, 5071–5082. 10.1167/iovs.10-5289 20484586 PMC3066595

[B49] Trost A. Motloch K. Bruckner D. Schroedl F. Bogner B. Kaser-Eichberger A.. (2015). Time-dependent retinal ganglion cell loss, microglial activation and blood-retina-barrier tightness in an acute model of ocular hypertension. Exp. Eye Res. 136, 59–71. 10.1016/j.exer.2015.05.010 26001526

[B50] Urushitani M. Nakamizo T. Inoue R. Sawada H. Kihara T. Honda K.. (2001). N-methyl-D-aspartate receptor-mediated mitochondrial Ca^2+^ overload in acute excitotoxic motor neuron death: a mechanism distinct from chronic neurotoxicity after Ca^2+^ influx. J. Neurosci. Res. 63, 377–387. 10.1002/1097-4547(20010301)63:5<377::aid-jnr1032>3.0.co;2-# 11223912

[B51] Villarreal A. Aviles Reyes R. X. Angelo M. F. Reines A. G. Ramos A. J. (2011). S100B alters neuronal survival and dendrite extension via RAGE-mediated NF-kappaB signaling. J. Neurochem. 117, 321–332. 10.1111/j.1471-4159.2011.07207.x 21291473

[B52] Villarreal A. Seoane R. González Torres A. Rosciszewski G. Angelo M. F. Rossi A.. (2014). S100B protein activates a RAGE-dependent autocrine loop in astrocytes: implications for its role in the propagation of reactive gliosis. J. Neurochem. 131, 190–205. 10.1111/jnc.12790 24923428

[B53] Wang X. Ng Y. K. Tay S. S. (2005). Factors contributing to neuronal degeneration in retinas of experimental glaucomatous rats. J. Neurosci. Res. 82, 674–689. 10.1002/jnr.20679 16273539

[B54] Yang J. Tezel G. Patil R. V. Romano C. Wax M. B. (2001). Serum autoantibody against glutathione S-transferase in patients with glaucoma. Invest. Ophthalmol. Vis. Sci. 42, 1273–1276. 11328739

[B55] Yu S. W. Andrabi S. A. Wang H. Kim N. S. Poirier G. G. Dawson T. M.. (2006). Apoptosis-inducing factor mediates poly(ADP-ribose) (PAR) polymer-induced cell death. Proc. Natl. Acad. Sci. U S A 103, 18314–18319. 10.1073/pnas.0606528103 17116881 PMC1838748

