# Supplementary figures and images for: Intravitreal S100B Injection Leads to Progressive Glaucoma Like Damage in Retina and Optic Nerve
Source: Front Cell Neurosci. 2018 Sep 26;12:312. doi: 10.3389/fncel.2018.00312 (PMC6169322; doi:10.3389/fncel.2018.00312)

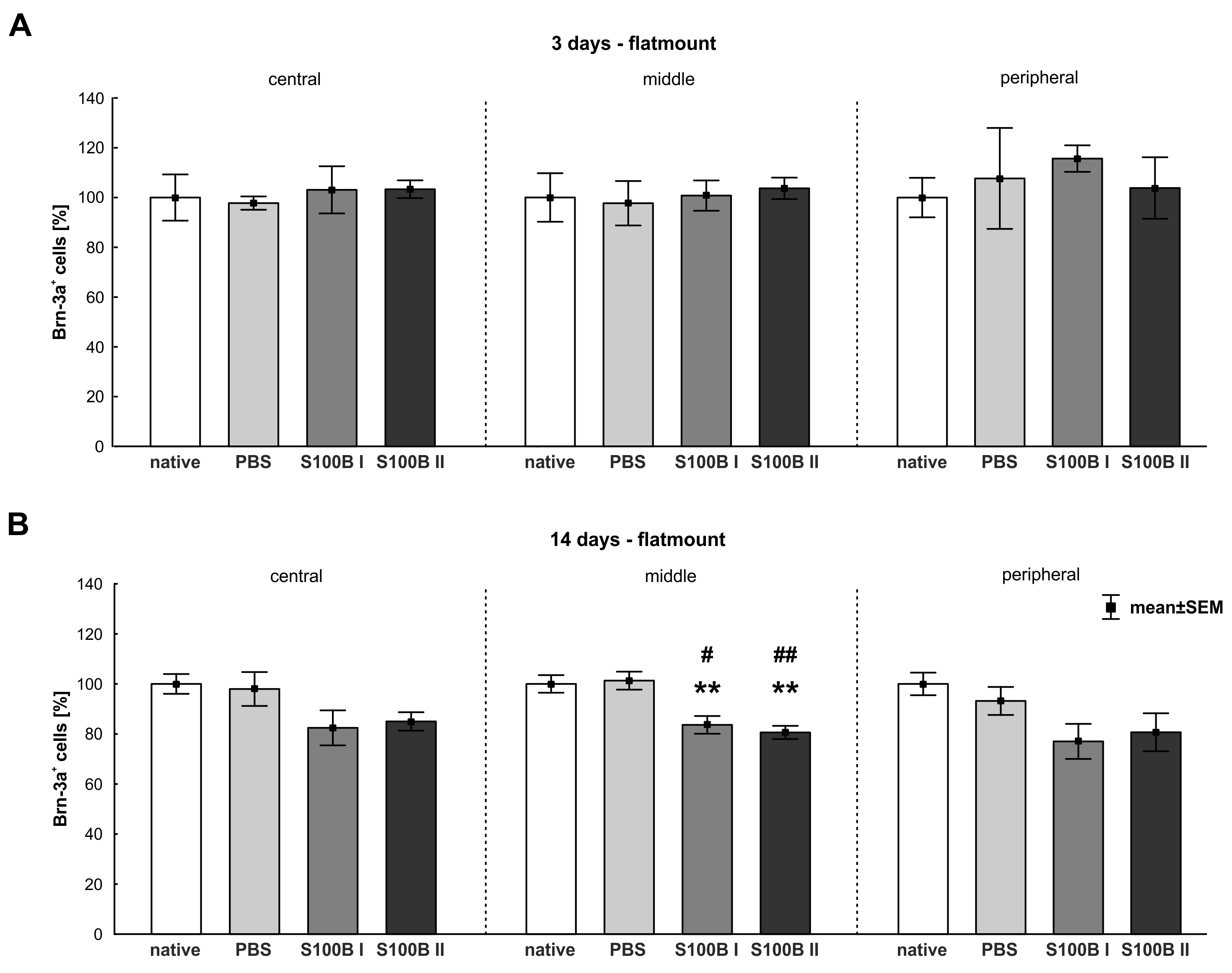

Supplement: FIGURE S1 — RGC density in different flatmount regions. (A) At day 3, the RGC numbers in all three regions, peripheral, middle and central of S100B flatmounts were comparable to the PBS and native group. (B) At day 14, RGC loss was noted in the middle part of the retina compared to the PBS (S100B I: p = 0.009, S100B II: p = 0.002) and native group (S100B I: p = 0.02, S100B II: p = 0.002). **p < 0.01 (compared to PBS group), #p < 0.05, ##p < 0.01 (compared to native group). [file Image_1.TIF]
